# PET Quantification of Cerebral Oxygen Metabolism in Small Animals

**DOI:** 10.1155/2014/159103

**Published:** 2014-08-17

**Authors:** Takashi Temma, Kazuhiro Koshino, Tetsuaki Moriguchi, Jun-ichiro Enmi, Hidehiro Iida

**Affiliations:** Department of Investigative Radiology, National Cerebral and Cardiovascular Center Research Institute, 5-7-1 Fujishiro-dai, Suita, Osaka 565-8565, Japan

## Abstract

Understanding cerebral oxygen metabolism is of great importance in both clinical diagnosis and animal experiments because oxygen is a fundamental source of brain energy and supports brain functional activities. Since small animals such as rats are widely used to study various diseases including cerebral ischemia, cerebrovascular diseases, and neurodegenerative diseases, the development of a noninvasive *in vivo* measurement method of cerebral oxygen metabolic parameters such as oxygen extraction fraction (OEF) and cerebral metabolic rate of oxygen (CMRO_2_) as well as cerebral blood flow (CBF) and cerebral blood volume (CBV) has been a priority. Although positron emission tomography (PET) with ^15^O labeled gas tracers has been recognized as a powerful way to evaluate cerebral oxygen metabolism in humans, this method could not be applied to rats due to technical problems and there were no reports of PET measurement of cerebral oxygen metabolism in rats until an ^15^O-O_2_ injection method was developed a decade ago. Herein, we introduce an intravenous administration method using two types of injectable ^15^O-O_2_ and an ^15^O-O_2_ gas inhalation method through an airway placed in the trachea, which enables oxygen metabolism measurements in rats.

## 1. Introduction

Since cerebral blood flow (CBF) and oxygen metabolism are fundamental for brain activity, the* in vivo *measurement of CBF, oxygen extraction fraction (OEF), and cerebral metabolic rate of oxygen (CMRO_2_) is of great importance in clinical diagnosis and for animal experiments. In particular, small animals such as mice and rats are widely used for research in a variety of diseases such as cerebral ischemia [1], dementia [2], Alzheimer's disease [3], and neurodegenerative diseases [4]. Small animals are also useful for the elucidation of glial function in pathological conditions [5] and for understanding the functional relationship between the brain and peripheral organs [6]. Therefore, the development of a noninvasive* in vivo* measurement method of such cerebral metabolic parameters in small animals has been eagerly sought.

Positron emitters, such as ^18^F, ^15^O, ^11^C, and ^13^N, emit positrons (*β* ^+^) from which pairs of photons are detected by positron emission tomography (PET) to generate reconstructed images. This involves several corrections for random coincidence events, dead time count losses, detector inhomogeneity, photon attenuation, and scatter, among others. The annihilation radiation can noninvasively transmit through biological tissues. Thus positron-labeled compounds are used in combination with PET imaging to obtain biological information of living systems in research and clinical settings. For instance, ^15^O labeled O_2_ gas PET has been used to estimate cerebral oxygen metabolism in patients for diagnostic purposes since the 1970s [7–12]. Although the ^15^O-O_2_ gas PET technique also attracted researchers for the evaluation of cerebral oxygen metabolism in small animals, it was applied unsuccessfully due to technical challenges until the 1990s. To overcome these challenges, several methodological inventions have been tried, which have facilitated the evaluation of CMRO_2_ and OEF in small animals in the current research setting.

Herein, we introduce an intravenous administration method using injectable ^15^O-O_2_ and an inhalation method of ^15^O-O_2_ gas, both of which can measure CMRO_2_ and OEF with PET in living rats under anesthesia.

## 2. Intravenous Administration Method

**Figure 1:**
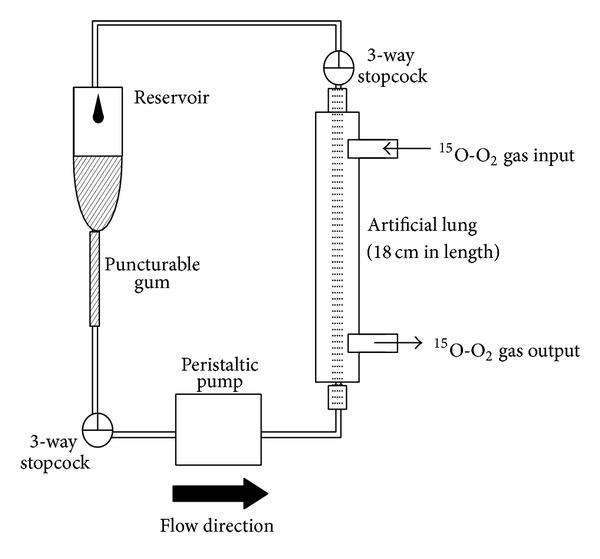
Injectable ^15^O-O_2_ preparation system using an 18 cm long artificial lung. The length of the artificial lung was 6 cm in the original report [13] and was changed to 18 cm in the latter studies for improvement of labeling efficiency [14, 15, 17].

**Figure 2:**
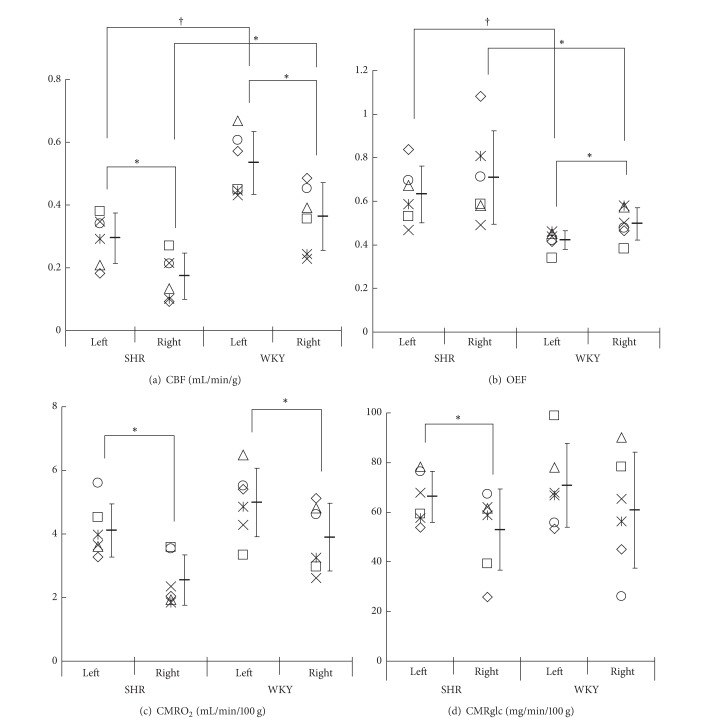
(Figure  1 in [15]) Quantitative values of CBF (a), OEF (b), CMRO_2_ (c), and cerebral metabolic rate of glucose (CMRglc) (d). PET with ^15^O-water and injectable ^15^O-O_2_ and an* ex vivo* autoradiography with ^18^F-FDG were performed one hour after the onset of a right middle cerebral artery occlusion using spontaneously hypertensive rats (SHR) and Wistar Kyoto rats (WKY). CBF, OEF, and CMRO_2_ were obtained from PET and CMRglc was obtained from ARG. Each of the six marks indicates the hemispheric average of 4 slices in an individual. Bar-shaped marks show the average and the error bars represent SD. Significant differences between hemispheres and between SHR and WKY were determined using the Wilcoxon signed rank test, *P < 0.05, and the Mann-Whitney *U* test, *P < 0.05, ^†^ *P* < 0.01.

Although the importance of evaluating cerebral oxygen metabolism in small animals has been recognized, application of the inhalation method using ^15^O-O_2_ gas in small animals could not be performed due to technical issues such as the potential influence of high radioactivity in the inhalation tube on the rat brain data acquisition. To overcome this situation, Magata et al. first developed an ^15^O-O_2_ injection method, which made rat OEF measurement possible using PET [13]. They collected blood from several rats and labeled the blood with ^15^O-O_2_ gas using an artificial lung (Figure 1). After 10 minutes of ^15^O-O_2_ uptake into the red blood cells, they had ^15^O labeled blood (72 MBq/mL) to use as an injectable for intravenous administration into normal rats for PET imaging. In fact, they performed two serial PET scans with ^15^O-water and injectable ^15^O-O_2_ and obtained 44 ± 4.5 mL/min/100 g of CBF and 0.54 ± 0.11 of OEF in normal rats under pentobarbital anesthesia. Subsequently, the same group evaluated the utility of the injectable ^15^O-O_2_ PET system using brain infarction rats [14], hypertensive rats [15], and normal monkeys [16]. The results indicated that the injectable ^15^O-O_2_ PET system could provide information on cerebral oxygen metabolism under normal and pathological conditions in rats as well as in larger animals. In particular, using the injectable ^15^O-O_2_ PET technique in spontaneously hypertensive rats (SHR), this research group clearly demonstrated that hypertension could intensify cerebral metabolic disturbances during the acute phase after the onset of stroke (Figure 2 [15]). This same group also applied the ^15^O-O_2_ injection technique to miniature pigs to evaluate myocardial oxygen metabolism, which was also considered to be a difficult target for evaluation by ^15^O-O_2_ gas inhalation because of the existence of radioactivity spillover from the gas volume in the lung to the myocardium due to limited spatial resolution [17]. Although the blood-based injectable ^15^O-O_2_ system provided a strong option that enabled oxygen metabolism measurement in small animals under normal and pathological conditions, some drawbacks were addressed for further applications. Namely, the blood-based injectable ^15^O-O_2_ system required that additional rats be sacrificed for blood collection and there was a possibility that the biological characteristics of the blood components might be damaged during the preparation process.

**Figure 3:**
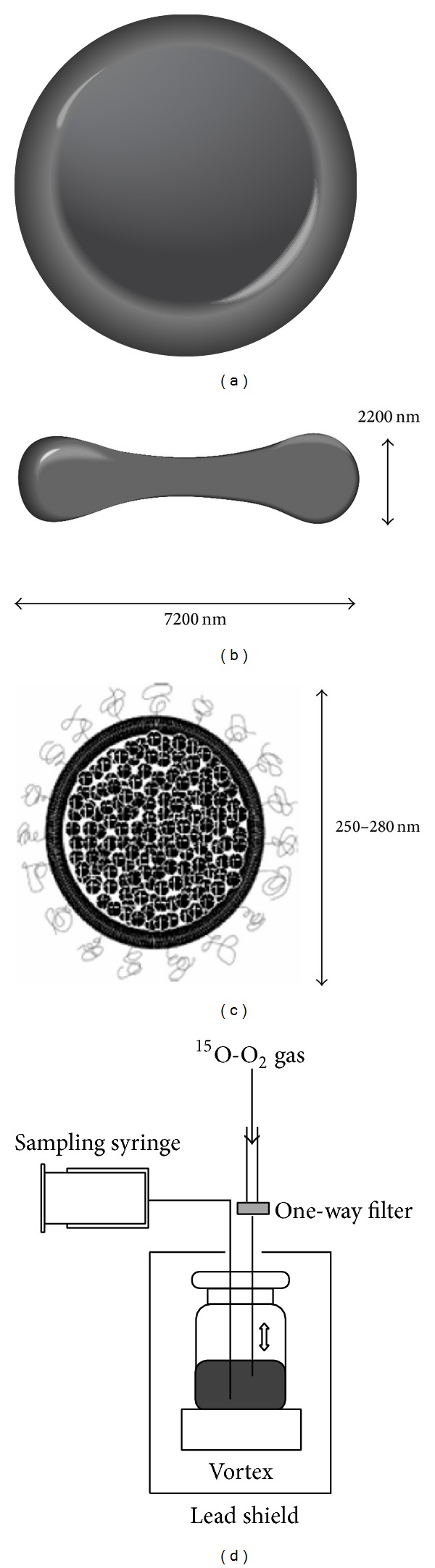
A schematic diagram of the ^15^O-O_2_-HbV preparation system. Normal human red blood cells (RBC) (a, b), hemoglobin-vesicle (HbV) structure (c) with shape and approximate diameters, and the final labeling setup with a lead shield for injectable ^15^O-O_2_-HbV preparation (d) are shown (courtesy of Dr. Kiyono, University of Fukui, Fukui, Japan).

Tiwari et al. then reported on a different injectable ^15^O-O_2_ system using hemoglobin-containing vesicles (HbV) to overcome these problems (Figure 3) [18]. The HbV, originally developed as an alternative oxygen carrier [19], was a liposome (about 300 nm in diameter) consisting of 1,2-dipalmitoyl-*sn*-glycero-3-phosphatidylcholine (DPPC), cholesterol, and 1,2-dipalmitoyl-*sn*-glycero-3-phosphoglycerol (DPPG) (5/5/1 at a molar ratio) and containing 10.8 g/dL hemoglobin molecules. The authors tested the feasibility of the HbV as an ^15^O-oxygen carrier, optimized a preparation system to obtain ^15^O-O_2_-HbV with a high labeling yield, and performed a PET study in normal rats after intravenous administration of ^15^O-O_2_-HbV. As a result, they achieved optimization of the labeling procedure using a direct bubbling method of ^15^O-O_2_ gas into the HbV solution containing L-cysteine using a vortex. They obtained 214 ± 7.8  MBq/mL ^15^O-O_2_-HbV, which is about 3-fold higher than the previous blood-based injectable ^15^O-O_2_ [13]. They also measured CBF, OEF, and CMRO_2_ values using the ^15^O-O_2_-HbV with PET imaging in normal rats. The same research group from the University of Fukui proceeded to lessen the invasiveness of the ^15^O-O_2_ injection method in the next step. In fact, all of the manuscripts using the ^15^O-O_2_ injection method described above adopted continuous arterial blood sampling during the PET scans for estimation of the input function to analyze cerebral metabolic parameters [13–16, 18]. Since the total volume of blood sampling is limited in small animals such as rats, they applied a steady-state method they originally developed for CBF measurement using ^15^O-water PET in rats [20] to the ^15^O-O_2_-HbV PET to decrease the injection and blood sampling volumes [21]. They prepared ^15^O-water, ^15^O-O_2_-HbV, and ^15^O-CO-HbV obtained in a similar manner as the ^15^O-O_2_-HbV, and PET scans were performed with continuous intravenous administration of ^15^O-CO-HbV, ^15^O-water, and ^15^O-O_2_-HbV through a multiprogrammed syringe pump with gradual changes in the injection speed. They reported that the injection and sampling blood volumes were 1.65 and 0.65 mL in ^15^O-water PET and 1.65 and 1.40 mL in ^15^O-O_2_-HbV PET, respectively, and achieved the measurement of CBF, OEF, CMRO_2_, and cerebral blood volume (CBV) values in several cerebral regions using a high resolution PET system (SHR-41000; Hamamatsu Photonics, Hamamatsu, Japan). In addition, the usefulness of the steady-state method was confirmed in a rat model of brain infarction. As such, in combination with the improvement in small animal PET systems and experimental procedures, the ^15^O-O_2_ intravenous administration method made possible cerebral oxygen metabolism measurement of rats in normal and pathological conditions, with minimal invasiveness.

## 3. Inhalation Method

Aside from the intravenous administration method, researchers have also tried to develop an ^15^O-O_2_ gas inhalation method for small animals such as rats. Yee et al. first performed a micro-PET experiment using normal rats with briefly inhaled ^15^O-O_2_ gas [22]. In this report, the authors applied the one-step method using single inhalation of ^15^O-O_2_ gas [23] to rats, and the ^15^O-O_2_ gas contained in a syringe was administered by a bolus insufflation into the lung through a cannula surgically placed in the trachea. In addition, they omitted arterial blood sampling in consideration of the limited blood volume of rats. Instead, for the estimation of input function, the field of view (FOV) of a PET scan was positioned to cover the brain and the heart at the same time. The time activity curve data from the heart was corrected using the volume ratio of the pure arterial space inside the ROI as the arterial input function [24]. As a result, 5.00 ± 0.36 mL/min/100 g of CMRO_2_ was calculated in 10 normal rats under *α*-chloralose anesthesia with continuous infusion. The study was successfully performed to achieve rat CMRO_2_ measurement with only one PET scan and without arterial blood sampling; however, a tracheotomy for tracer administration, animal size restriction for simultaneous brain-heart scan, and poor signal to noise ratio were mentioned as limitations.

Recently, Watabe et al. reported the application of a steady-state ^15^O-O_2_ gas inhalation method for normal rats [25]. Namely, they performed a tracheotomy and placed a flexible tube into the trachea to serve as an administration route for the ^15^O-gas tracers. They performed three serial PET scans using ^15^O-CO_2_, ^15^O-O_2_, and ^15^O-CO gas, respectively, and measured CBF, OEF, CMRO_2_, and CBV values in the normal brains of rats under anesthesia according to the original ^15^O gas steady-state inhalation method used in clinical settings [26–28]. A clinical PET camera (Headtome-V PET scanner; Shimadzu Corp.) was used and the feasibility of using the camera for small animal studies was evaluated by phantom experiments. After precise evaluation of partial volume effects, scatter correction from the high radioactivity in the pleural cavity, and application of a cross-calibration factor, the authors succeeded in obtaining quantitative and comparable values and functional images of CBF, OEF, CMRO_2_, and CBV in normal rats. In addition, they tested the applicability of the method to a small number of ischemia model rats (*n* = 2) and successfully showed decreased CBF and CMRO_2_ values and increased OEF value in the ipsilateral hemisphere. The total time was about 73 min for the entire PET experiment in each rat. The results clearly indicated that the steady-state ^15^O-gas inhalation method used in clinical settings could be applied to rats with consideration of the appropriate care to avoid possible errors. However, tracheotomy was still required for gas tracer administration and the rats underwent arterial blood sampling during the PET scan, which might be considered a limitation in the above study.

**Figure 4:**
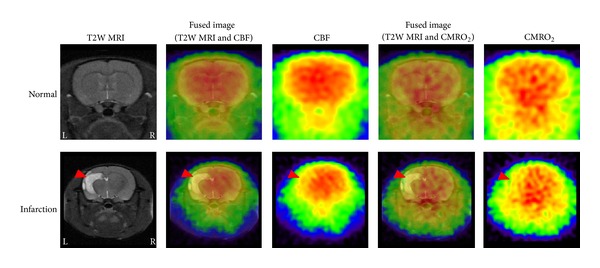
Functional images (“pseudo” CBF and CMRO_2_) of normal and infarction rat brains (Wistar rats, male, 8 weeks old). T2 weighted MR images are shown as a position reference. PET scans were performed during continuous administration of ^15^O-CO_2_ and ^15^O-O_2_ gases by spontaneous respiration of rats under isoflurane anesthesia.

On this basis, we are now developing an ^15^O gas administration technique that uses the spontaneous respiration of rats under isoflurane anesthesia for micro-PET measurement of cerebral metabolic function without arterial blood sampling. As shown in Figure 4 (unpublished data), we can provide “pseudo” functional images of a rat brain under both normal and pathological conditions. We expect to successfully perform this technique in the near future.

Finally, regardless of the administration route of ^15^O-O_2_, recirculating ^15^O labeled water, which is a metabolic product of ^15^O-O_2_, should be taken into consideration for estimating quantitative CMRO_2_ and OEF in small animals. The recirculating ^15^O-water could have a crucial impact on these parameters due to more rapid appearance after ^15^O-O_2_ administration in small animals than in humans. In fact, most of the studies described above measured the contribution of recirculating ^15^O-water as an input function by separating the plasma from the whole blood samples [13–15, 21, 25]. However, this procedure requires repetitive blood sampling during a PET study, which may alter physiological function due to the limited total blood volume in small animals. Recently, an alternative approach has been applied, in which the time activity curve of recirculating ^15^O-water could be predicted from a whole blood radioactivity concentration curve by modeling the kinetics of the metabolic process of oxygen molecules in the whole body [29]. Thus, the labor intensive procedure of frequent arterial blood sampling with centrifugation can be avoided, making the protocol applicable to many studies using clinical patients as well as experimental animals. It is of note that this method was shown to be applicable to a wide range of species from human to rats. Therefore, using the simplified method to predict the contribution of recirculating ^15^O-water, in combination with less invasive techniques to obtain the time activity curve such as an online scintillation detector coupled to an arteriovenous shunt [30] or ROI analysis of the cardiac ventricle in PET images [22], the ^15^O PET technique could be more widely applied to small animals under a broad range of conditions.

## 4. Conclusion

Since oxygen is a key molecule for energy production in living brains, the measurement of cerebral oxygen metabolism is important to understand brain function in normal and pathological conditions. With some technological innovations including the development of injectable ^15^O-O_2_ preparations and the successful application of an ^15^O-O_2_ gas inhalation method with appropriate corrections, measurement of cerebral oxygen metabolism (OEF and CMRO_2_) has become possible in living rats, as compared to the difficult challenges faced more than a decade ago. However, there are several issues that remain unresolved for the ideal achievement of noninvasive quantification of OEF and CMRO_2_ in living rats by PET using ^15^O gas tracers; these include tracheotomy, arterial blood sampling, and long experimental time. In contrast, the total examination time in clinical settings has been dramatically reduced from more than 40 minutes [31] to about 10 minutes by recent technical innovations [9, 32]. Therefore, experiments involving small animal models would also benefit from further methodological progress including faster and less invasive measurement (e.g., ^15^O gas administration by spontaneous respiration, input function estimation from the heart or large arteries) with improvement of resolution and sensitivity by dedicated PET scanners for small animals and the development of a fully automated rapid measurement system for animal ^15^O gas experiments. With such innovation, the ^15^O PET technique could be more widely applied to studies in model animals including not only ischemia and infarction but also neurodegenerative and psychiatric diseases.
